# Post Natal Depression Prevalence: A Comparison Between Pakistan and the United Kingdom

**DOI:** 10.1192/bjo.2026.11594

**Published:** 2026-06-30

**Authors:** Rabia Rehman

**Affiliations:** King's College London, London, United Kingdom

## Abstract

**Aims:**

Maternal health encompasses wellbeing before, during, and after pregnancy, with thepostnatal period critical for physical and psychological recovery. Prevalence of postnatal depression (PND) is higher in Pakistan (28-63%) compared to the United Kingdom (UK) (15-20%).

This study aims to compare PND prevalence among Pakistanis, British Pakistanis, and the wider UK population in line with three objectives:

1) Exploring cultural factors influencing PND

2) Identifying factors driving higher prevalence in Pakistan

3) Evaluating support structures while proposing culturally sensitive interventions

**Methods:**

This study combines a comparative narrative review of secondary sources with primary qualitative input. Epidemiological data and government reports were synthesised to compare prevalence and healthcare systems. Additionally, clinician perspectives were collected as a podcast providing cultural context. Short films were also interpreted to illustrate societal attitudes towards maternal mental health and explore lived experiences.

**Results:**

PND is influenced by the biopsychosocial model with cultural stigma, somatisation, and barriers to care being key drivers in Pakistan. Severe workforce shortages (0.19 psychiatrists per 100,000 compared to 11 per 100,000 in South London), high home birth rates (63%), and lack of routine postnatal checks exacerbate risks. Traditional practices like Chilla offer post-partum support but should be supplemented with structured services similarto the UK which provide postnatal checks and specialist mother and baby units (MBUs), although access remains unequal across the UK. Funding disparities persist with Pakistan allocating only 0.4% of its health budget to mental health compared to 8.1% by NHS England. These systemic gaps highlight the need for integrated and culturally sensitive approaches that address both clinical and societal factors of care.

**Conclusion:**

Improving maternal mental health requires strategies combining psychiatry, public health, and cultural awareness. Recommendations include tailored cultural psychiatry training for UK professionals, increased funding for maternal mental health in Pakistan, and stigma reduction campaigns. Bridging clinical care and lived experience can reduce inequalities and enhance maternal wellbeing globally.

